# Digitalization within the MME study program – teaching and assessment of communicative and interprofessional skills in the Heidelberg module via video conference together with a virtual OSCE course

**DOI:** 10.3205/zma001381

**Published:** 2020-12-03

**Authors:** Saskia Veronika Pante, Michael Weiler, Bernhard Steinweg, Anne Herrmann-Werner, Christian Brünahl, Maryna Gornostayeva, Konstantin Brass, Anna Mutschler, Andrea Schaal-Ardicoglu, Stefan Wagener, Andreas Möltner, Jana Jünger

**Affiliations:** 1 University Heidelberg, Medical Faculty, MME study program, Heidelberg, Germany; 2 University Heidelberg, Medical Faculty, Center of Excellence for Assessment in Medicine, Heidelberg, Germany; 3 University Hospital of Bonn, Office of the Dean of Studies, Medical Faculty, Bonn, Germany; 4 University Hospital of Tübingen, Department of Psychosomatic Medicine and Psychotherapy, Tübingen, Germany; 5 Institute of Medical and Pharmaceutical Proficiency Assessment, Mainz, Germany; 6 Institute for Communication and Assessment Research, Heidelberg, Germany; 7 University Heidelberg, Medical Faculty, Medi-KIT, Heidelberg, Germany

**Keywords:** Master of Medical Education, OSCE, digitalization

## Abstract

**Background: **Due to the corona pandemic, we conducted the Heidelberg module of the Master of Medical Education (MME) study program, which focuses on teaching and assessment of communicative and interpofessional skills, digitally for the first time.

**Method:** We outsourced the teaching to a pre-module phase in the weeks upfront. During the module week, the lecturers picked up again and deepened the topics and the participants created, revised and simulated a virtual OSCE course.

**Results/Conclusion: **Evaluation and reflection of the module showed that the digital implementation including an OSCE examination can be an appropriate alternative to a classroom-based training. However, important elements of the MME program that provide networking possibilities and personal exchange can only be replicated in the digital environment to a limited extent. In the future, sensibly applied digital components can be used to enrich the study program.

## Zusammenfassung

**Hintergrund: **Bedingt durch die Corona-Pandemie wurde das Heidelberger Modul des Studiengangs Master of Medical Education, das seinen Schwerpunkt auf Lehren und Prüfen kommunikativer und interpofessioneller Kompetenzen legt, erstmals digital durchgeführt.

**Methode:** Die Lehre wurde in eine Prä-Modulphase in die Vorwochen ausgelagert. In der Modulwoche wurden die Themen erneut aufgegriffen und vertieft sowie ein virtueller OSCE Parcours durch die Teilnehmer*innen erstellt, revidiert und simuliert.

**Ergebnisse/Schlussfolgerung: **Evaluation und Reflexion des Moduls ergaben, dass die digitale Durchführung samt einer OSCE Prüfung eine angemessene Alternative zu einer Präsenzveranstaltung darstellen kann. Allerdings können wichtige Elemente des Studiengangs, die zum Netzwerken und persönlichen Austausch dienen, digital nur bedingt repliziert werden. Künftig können sinnvoll eingesetzte digitale Anteile das Studium bereichern.

## 1. Background

The goal of the Master of Medical Education (MME) Germany study program is to train multipliers and leaders for teaching and examination [1]. The Heidelberg module focuses on communication with patients and interprofessional cooperation by interactively training the role of the professional teacher with simulation persons (SPs) and by creating, revising, and testing the appropriate station for an Objective Structured Clinical Examination (OSCE) course in oral-practical examinations in the sense of *constructive alignment*. In the wake of the corona pandemic, we held the module with 27 participants online for the first time. The objective was to investigate whether the implementation of a module aimed at interaction can be successfully transferred into a digital environment and whether initial findings can be confirmed that virtual OSCEs promote the development of telemedical skills [2], [3], [4]. 

## 2. Virtual module

We used a video conference system conforming to the data protection regulations for the digital implementation [https://www.urz.uni-heidelberg.de/de/heiconf]. One month before the start of the module an introduction took place with information about the implementation and topics of the module. We reduced the attendance time during the module week from 45 to approx. 25 hours. 25 hours. The remaining time was outsourced to the weeks beforehand as a *flipped classroom* (pre-module phase) in order to enable the preparation according to the participants own schedule and to reduce the screen time during the attendance phase. The lecturers used medical didactic principles to reflect on which lectures and tutorials were offered in advance and which refreshers were placed in the module. In addition, we provided individual appointments for SP role acceptance. We used a cloud system [https://www.urz.uni-heidelberg.de/de/heibox] in order to provide the participants all learning content online and to enable them to jointly work together on files. During the module week, the prepared learning content was recapitulated in compressed form as core aspects and then discussed. In addition, the module team jointly reviewed all materials, such as work orders, instructions, and guidelines and adapted them in their comprehensiveness and clarity for the digital implementation. This was done in order to address the reduced physical responsiveness of the module team.

## 3. Virtual OSCE

**Figure 1:**
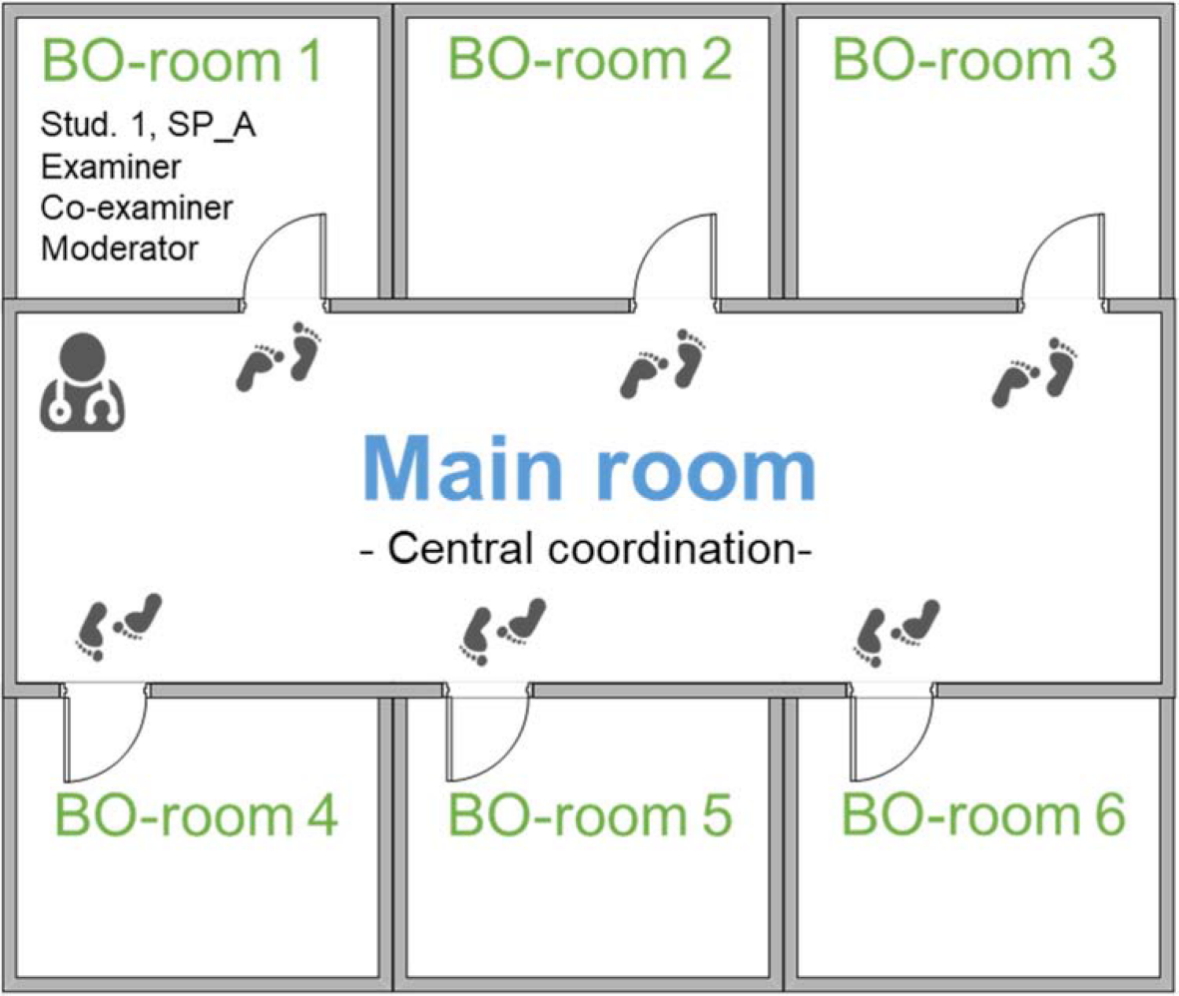
Digital OSCE. Starting from the main room of the video conferencing tool heiCONF, the students and examiners entered, according to their individual rotation plans, the breakout (BO)-rooms. Students and simulation persons (SPs) added a unique identifier to their username for reasons of clarity. During the OSCE course, the SPs and the moderators always stayed within the same BO-rooms. The moderators had several tasks to fulfill including checking the individual’s presence (after consultation with the coordination who remained in main room), starting the assessment and ending it after exactly eight minutes, presenting the vignette as well as the task, and recording of the assessment. During the actual exam the main examiner, the examinee, and the SP had their webcam switched on. As an illustrative example, the occupancy of BO-room 1 is shown.

We gave the participants the preparatory task of designing a digital lecture on the topic of communication and interprofessionalism including a suitable OSCE station, which was subjected to various review and feedback processes in the pre-module phase and module week. The participants specified and tested the roles of the SPs in a multi-stage process. The lecture and OSCE should preferably be aimed at digital contexts and included, for example, an online consultation hour and an interprofessional patient handover. The lecture and OSCE exams were conducted synchronously online together with medical students, who we recruited by e-mail. These students took on the role of students/examinees, while the MME participants acted as lecturers/examiners. The OSCE was structured as follows:

- From the main room we set up 6 BreakOut (BO) rooms according to the 6-station course, into which the participants changed independently in accordance with their rotation plan (see figure 1 (Fig. 1)).
- The evaluation of the different OSCE stations was done by means of tablets [https://www.ucan-assess.org/tools/]. Additionally, the corresponding paper version was available. Depending on the station, the checklist used item lists and/or global ratings.
- After 12 minutes the software automatically terminated the BO rooms and we started a new examination round from the main room.
- At the end of the course, all participating groups collected feedback on all stations in the sense of a 360° reflection.

As a follow-up task, we asked the participants to reflect on the lecture and OSCE stations in terms of constructive alignment and to revise them on the basis of the obtained feedback. Optionally, we offered the participants the possibility to test the OSCE stations in a real setting. 

## 4. Evaluation

**Figure 2:**
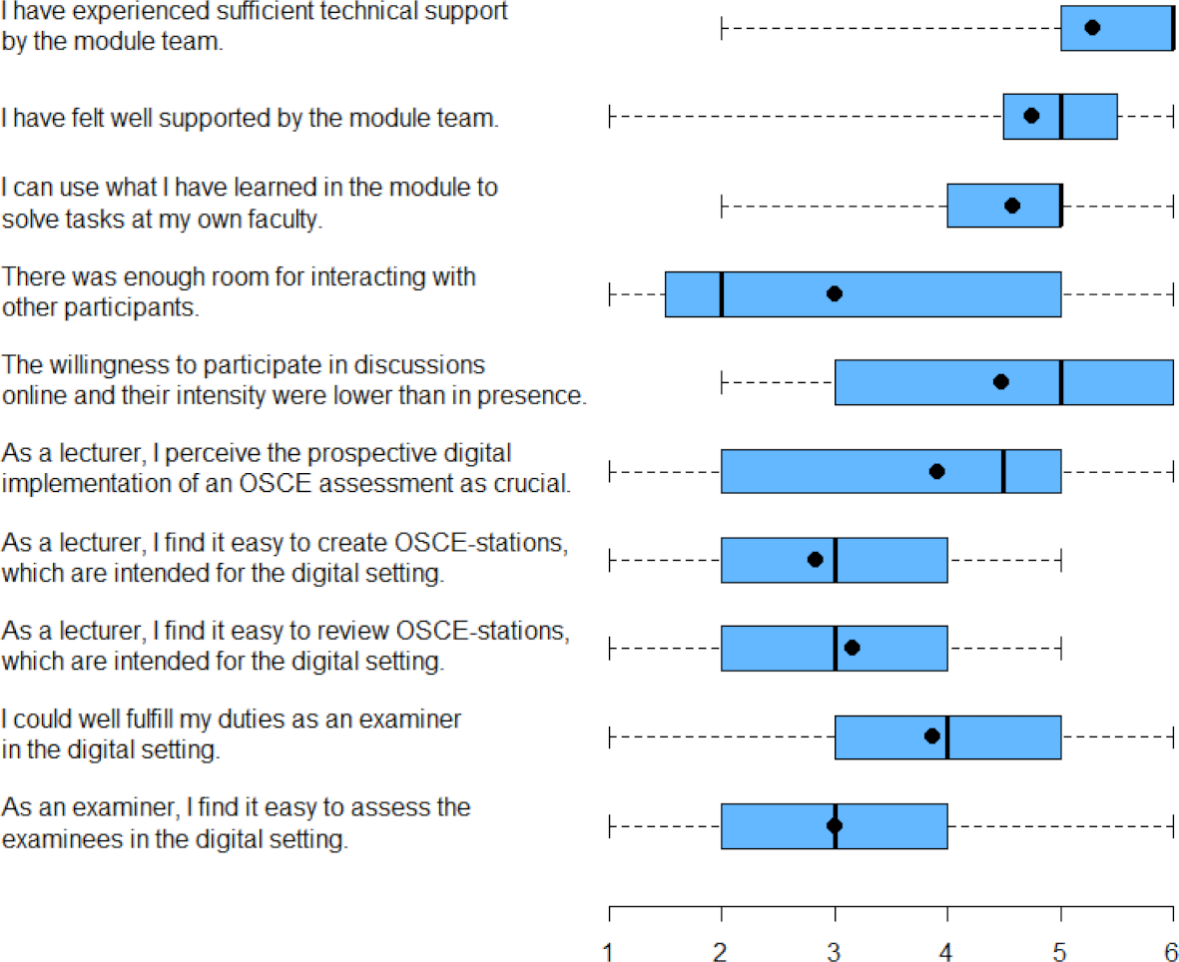
Samples from the module evaluation by the MME participants. The figure shows the distribution of the answers as a boxplot (“skeletal box and whiskers plot”). Scale value 1 refers to “I do not agree”, while scale value 6 pertains to “I do fully agree”. The “box” encompasses the area between the lower and upper quartile. The median is displayed as a vertical line and the arithmetic mean is delineated as a dot. The difference between the upper and lower quartiles resembles the interquartile range (IQR). The “whiskers” range from the minimal to the maximal value.

At the end of the module week the participants took part in an online evaluation generated by EvaSys, which included 76 questions (standardized and free text), out of which 21 were specifically related to the implementation of the online module. The results of the evaluation (see figure 2 (Fig. 2)) showed that the content and technical implementation of the module were generally evaluated as successful, instructive, and transferable to the own faculty. The participants also perceived the individual time allocation in the pre-module phase and the lack of travel time as positive. Besides the high organizational effort, additional (technical) support may often be required. At the same time, participation in the module from the clinical or private workplace may lead to a distraction of the participant. The participants cite limited discussion and networking possibilities in the interdisciplinary and interprofessional group as the most significant constraint in the virtual setting. With regard to the virtual OSCE examination, the participants state that the creation and implementation of the exam were not easy for them, but that they consider the digital implementation to be important in the future (see figure 2 (Fig. 2)). For the professionalization of virtual OSCEs (especially summative ones) a neutral video background and appropriate clothing is also necessary. In addition, it is also essential to adjust the criteria for testing communicative skills, including non-verbal behavior, and to clarify legal issues (e.g., use of the Internet during the exam). Examination vignettes which are also used digitally in real life (e.g. telemedicine), are good to implement and important to train. 

## 5. Conclusion

As the evaluation highlighted, the virtual execution of the module including OSCE is well realizable. Especially with regard to telemedical communication stations, we can gain valuable experience. For the future, further strategies must be developed here by adapting sample solutions and SP training to the digital conditions, e.g. by including ratings of non-verbal communication in the checklists [5]. Technical equipment and operability of the electronic systems must also be guaranteed among all participants. Technical problems could be sufficiently counteracted by the training courses that are conducted in advance with all participants and the administration accompanying the module. On the other hand, the personal exchange and group dynamics, which are highly valued in the course of studies, suffer – both are important factors for the learning success of the students. In regards to the upcoming digitalization processes in medical studies [6], [7], [8], combining attendance modules with digital components seem to be attractive for the future.

## Acknowledgements

The authors would like to thank Kerstin Lubik, Sofia Gelashvili and Liane Ásgeirsson as well as the members of the OSCE team of the IMPP, the simulation persons, and the students for their active support and good cooperation.

## Competing interests

The authors declare that they have no competing interests.
